# Investigating the Effects and Mechanisms of Combined Vitamin D and K Supplementation in Postmenopausal Women: An Up-to-Date Comprehensive Review of Clinical Studies

**DOI:** 10.3390/nu16142356

**Published:** 2024-07-20

**Authors:** Marius Emil Rusu, Galya Bigman, Alice S. Ryan, Daniela-Saveta Popa

**Affiliations:** 1 Department of Pharmaceutical Technology and Biopharmaceutics, Faculty of Pharmacy, Iuliu Hatieganu University of Medicine and Pharmacy, 400012 Cluj-Napoca, Romania; 2 Division of Gerontology, Department of Epidemiology and Public Health, University of Maryland School of Medicine, Baltimore, MD 21201, USA; 3 Baltimore Veterans Affairs Medical Center, Division of Gerontology, Geriatrics and Palliative Medicine, Department of Medicine, University of Maryland School of Medicine, Baltimore, MD 21201, USA; aryan@som.umaryland.edu; 4 Baltimore Geriatric Research, Education and Clinical Center, Veterans Affairs Maryland Health Care System, Baltimore, MD 21201, USA; 5 Department of Toxicology, Faculty of Pharmacy, Iuliu Hatieganu University of Medicine and Pharmacy, 400012 Cluj-Napoca, Romania; dpopa@umfcluj.ro

**Keywords:** vitamins, nutrients, aging, trials, age-related diseases, cardiovascular health, bone health, osteoporosis

## Abstract

Aging is a complex process and a significant risk factor for chronic diseases. Menopause, a component of aging in women, is associated with several important cardiometabolic conditions including metabolic syndrome, osteoporosis, and cardiovascular diseases. Menopausal women could benefit from preventative strategies that may decrease morbidity and mortality and improve their quality of life. Vitamins D and K are essential nutrients required for bone health, immune function, and reducing cardiovascular risks, yet their synergistic effect is less understood in aging women. This is the first comprehensive review to summarize the evidence found in randomized clinical trials of the beneficial effects of vitamin D and K co-treatment in postmenopausal women. In our literature search across key electronic databases such as Cochrane, PubMed, and Ovid, we identified 31 pertinent studies. Overall, significant findings indicate that the combined intake of vitamins D and K may positively affect cardiovascular and bone health in postmenopausal women, emphasizing the importance of maintaining a healthy diet rich in vegetables and fermented dairy products. Given the challenges in obtaining all necessary nutrients solely through the diet, vitamin D and K supplements are recommended for postmenopausal women to promote healthy aging and well-being.

## 1. Introduction

Aging is a complex process and an essential risk factor in the development and advancement of many chronic disorders [1]. Aging and the menopausal transition are associated with lower estrogen hormone levels, increased total body fat, abdominal fat mass, and intramuscular fat, as well as insulin resistance, a poor lipid profile, and reduced cardiovascular fitness [2,3,4,5,6]. Well-known longitudinal trials such as the Women’s Health Initiative are particularly important to better understand the prevention and control of common chronic diseases in postmenopausal women [7]. The pathogenesis of various age-related diseases includes the accumulation of reactive oxygen species (ROS) that lead to oxidative stress and chronic inflammation, major factors for cellular senescence [8].

Cardiovascular disease (CVD) is the leading cause of death in women. Yet, a recent report indicated that the CVD burden for women in the past decade has not reduced and is understudied [9]. The cardiometabolic changes during and after the menopausal transition were recently reviewed to provide a better understanding of the management of women at a higher risk of CVD [10]. Longitudinal studies clearly show that women at menopause have an increased risk of developing CVD due to ovarian aging, sex hormone changes and body composition alterations, thereby stressing the importance of women’s health and providing early measures to reduce CVD risk [11]. Vascular calcification, where calcium is deposited in the vasculature, occurs with aging and contributes to atherosclerosis and the potential development of coronary heart disease, peripheral vascular disease, and stroke. A discussion of the progression of atherosclerosis is beyond the scope of this review but recent reviews have described the pathobiological mechanisms responsible for plaque development, progression and destabilization leading to major adverse cardiac events [12], and the role that sex may play in the development of atherosclerosis [13].

In addition to CVD, menopausal women are at risk of osteoporosis, which is a systemic bone disorder characterized by low bone mass, increased bone fragility and susceptibility to fracture. In fact, one in every three women over 50 years of age has an osteoporotic fracture during their lifetime [14]. Recent reviews of osteoporosis in postmenopausal women including the types of osteoporosis, its management and pharmacological treatments have provided specific insights regarding the loss of bone mass and susceptibility to bone fracture in women [15,16,17]. To maintain bone health in postmenopausal women, several important actions are recommended, including smoking cessation, physical activity, regular medical screening, and pharmacotherapy [18]. Various pharmacological treatments are available but many of them present adverse side effects. For example, some osteoporosis medications, such as bisphosphonates, denosumab, or romosozumab, may cause serious adverse events, including the development of atypical femoral fracture, jaw osteonecrosis, and CVD [19]. 

Hence, there is an increasing focus on alternative strategies to manage aging and prevent bone disorders and CVDs. Dietary strategies, nutrient-rich foods, and well-balanced diets containing adequate amounts of vegetables and fruits are suggested to lower the risk of pathologies associated with menopause and aging including cardiovascular and neurodegenerative diseases, metabolic complications, and osteoporosis [20,21,22,23]. Moreover, the intake of specific molecules may be a viable option to promote cardiovascular and bone health and reduce the disease burden in menopausal women [24,25].

The role of vitamin D (vitD) in cardiovascular and bone health has been summarized in terms of its effects on certain systems in postmenopausal women, with some debate [26,27]. VitD plays a key role in the regulation of calcium and phosphate homeostasis, and its metabolism involves numerous complex pathways [28]. Furthermore, vitamin D deficiency results in hyperparathyroidism, which increases bone resorption, reducing the bone mineral content, and increases the risk of fracture [29]. Likewise, but reviewed less, vitamin K (vitK) has been investigated for its role in altering bone metabolism [30,31]. The impact of vitK on bone health involves its absorption, metabolism and excretion, while the potential mechanisms associated with vitK’s effect on bone metabolism include its action on vitK-dependent proteins (VKDPs) to increase deposition and bone formation through various pathways, as recently reviewed [32]. The meta-analysis of Hao et al. noticed a significant inverse association between dietary vitamin K1 consumption and the risk of fractures [33], while Ma et al. indicated that vitK2 supplementation could promote bone mineralization, improve lumbar spine BMD, and increase bone strength, thereby reducing the risk of fractures [34]. Moreover, both preclinical and clinical studies have shown that the vitD and vitK association exerts a synergistic action by generating higher levels of bone anabolic markers and calcium deposits in osteoblasts [35], and a lower risk of hip fracture in elderly patients [36]. 

To the best of our knowledge, there are no reviews in the scientific literature that assess the effects of vitD and vitK co-treatment in postmenopausal women. Consequently, this is the first review to comprehensively analyze the data obtained in randomized controlled trials (RCTs) regarding vitD and vitK co-supplementation and their synergistic effects in reducing or preventing coronary artery calcification, promoting cardiovascular health, increasing the bone mineral density (BMD), and altering bone markers in postmenopausal women.

## 2. Methods

### 2.1. Search Strategy

Our narrative review includes randomized controlled parallel or crossover trial studies that examine the effects of vitD and vitK co-treatment in menopausal women. Through 30 April 2024, we conducted comprehensive electronic searches with the assistance of a professional librarian (please see acknowledgment for more details) to identify applicable publications. Our search spanned the major health databases, including Cochrane Library, PubMed, and Ovid, which are the preferred primary sources for identifying eligible literature, ensuring comprehensive coverage. In addition to these electronic searches, the authors systematically checked the references of the papers included in the review to identify additional articles.

To search the databases, the professional librarian utilized a combination of free-text words along with key concepts based on Medical Subject Headings (MeSH) for ‘RCT’, ‘randomized controlled clinical trials’, ‘vitamin D’, ‘vitamin K’, ‘menopause’, and ‘women/female’. Boolean operators (AND/OR) were employed to combine the words and search terms found in the title and/or abstract. For example, (((vitamin k[MeSH Terms]) AND (vitamin d[MeSH Terms])) AND (menopause[MeSH Terms])) AND ((woman[MeSH Terms]) OR (female[MeSH Terms])) were used. No date restrictions were imposed on the searches, and the search terms were intentionally broad and general to increase the likelihood of capturing potentially eligible studies.

### 2.2. Eligibility Criteria

Eligibility criteria were created by the corresponding authors (MER, GB). The selection process involved two team members (GB, MER) independently conducting a double-blind review of the titles and abstracts of all the retrieved references to identify applicable studies, following the de-duplication of search results, and all citations were stored in the reference manager database Zotero (Version 6.0.26, Corporation for Digital Scholarship, 2024, www.zotero.org). After the duplicate citations were removed, the screening and selection processes were as follows: (1) in the first round, articles were excluded based on their title; (2) in the second round, articles were excluded based on their abstract; and (3) in the final round, full-text papers were obtained and carefully assessed. The review eligibility criteria are described in Table 1. Only clinical trials were preselected, and of these, only RCTs were selected. We included only peer-reviewed published studies with at least an abstract presented in English. Studies were excluded if they did not present human research or if the participants were not experiencing menopause. The selected reviewed studies were confirmed by all the authors, and any disagreement was settled by discussion. 

### 2.3. Data Extraction Process

An electronic data extraction form was developed, encompassing the following predefined data fields: Author, Year of publication, Country, Population Age, Duration and Type of study, Intervention, Comparison/Diets, and main Outcomes. Two reviewers (DSP, MER) independently conducted a double-blind data extraction for each eligible study. 

**Figure 1:**
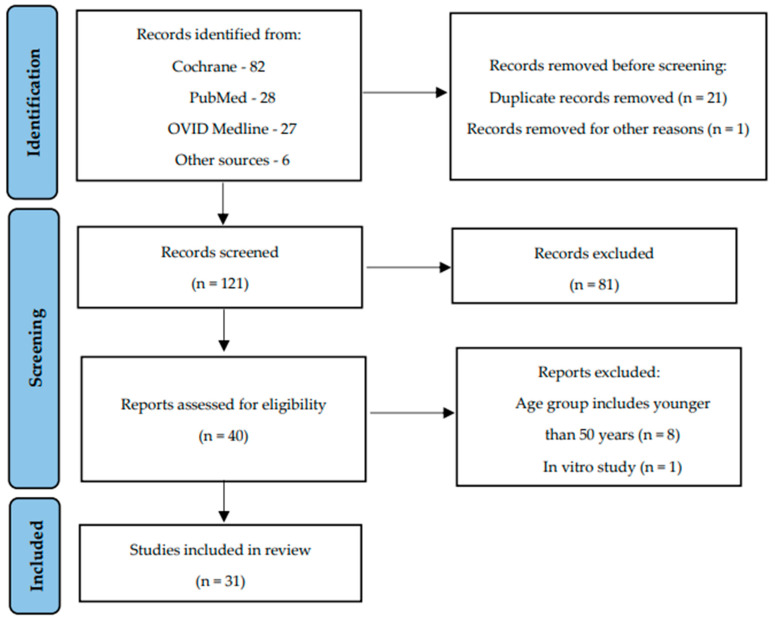
Flow chart illustrating the process of selecting studies.

A total of 143 articles were considered in the systematic search, with 22 duplicates removed (Figure 1). Subsequently, 121 studies and abstracts were reviewed from the relevant reference lists. After applying the eligibility criteria, a total of 31 articles were included in this review, of which six were related to cardiovascular health and 25 to bone health. It is worth mentioning that the methodology applied in this narrative review guarantees that the included articles are relevant, the findings are scientifically pertinent, and the data will be beneficial for future investigations.

## 3. Results and Discussion

### 3.1. Aging and Vitamins D and K

Menopausal women face distinct health challenges including declining estrogen levels, chronic inflammation, an altered lipid metabolism, and low levels of vitamins and minerals that have unfavorable implications for the cardiovascular system, bone health, carcinogenesis, or neurological function, along with increased risks of morbidity and mortality [37]. Moreover, osteoporosis and atherosclerosis, two processes connected to aging, may be related, as demonstrated by the association between a decreased BMD and an increased carotid intima–media thickness (CA-IMT) in postmenopausal women [38]. Growing evidence demonstrates that osteoporosis could be prevalent in atherosclerotic patients [39] and that interventions aimed at preventing one condition could positively influence others, ultimately slowing down the progression of aging and improving the quality of life [40,41].

Osteoporosis and vascular calcification (VC) are significant health issues that frequently coexist in the elderly population. Osteoporosis, the most common bone disorder that affects the aging population, is a cellular dysfunction between osteoblastic and osteoclastic cells characterized by lower bone formation and higher bone resorption, leading to bone loss, a reduction in bone mass, and an increased risk of bone fractures [42]. VC is defined as the ectopic accumulation of mineral matrix in the vessel wall [43]. Traditionally considered independent pathologies, recent findings have reported a possible direct correlation between these processes, defined as bone–vascular crosstalk [44]. Osteoporosis is linked to VC with common genetic and molecular signalling mechanisms, and common risk factors such as menopause, diabetes, obesity, and smoking [45]. A recent meta-analysis demonstrated that there is a correlation between the presence of abdominal aorta calcification accumulation, a quantifiable marker of vascular pathology, a decrease in BMD, and an increased relative risk of fracture at any site [46]. Furthermore, a low BMD is an independent predictor for early atherosclerotic CVD in women, and bone loss has been associated with the development and progression of VC and a higher risk of CVD [47,48]. The BMD of the lumbar spine has been inversely associated with the risk of coronary artery calcification (CAC) in adults with osteoporosis [49], while a higher BMD in the femoral neck and total proximal femur is a protective factor against coronary heart disease (CAD) [50]. Among the concepts proposed to explain the bone–vascular crosstalk are key risk factors such as hypertension, type 2 diabetes, oxidative stress, and chronic low-grade inflammation, or shared biomarkers including bone morphogenetic proteins, osteoprotegerin (OPG), and osteopontin [51]. New data show that the pro-inflammatory cytokines involved in many heart conditions and different CVDs such as tumor necrosis factor (TNF-α), interleukin-1 (IL-1), and IL-6 can also inhibit bone formation and induce bone resorption through the activation of osteoclasts [52].

There is evidence that low levels of vitD and vitK, key micronutrients, are associated with several age-related conditions, such as bone and cardiovascular diseases. An appropriate dietary intake or supplementation of these vitamins could prevent VC, enhance calcium absorption and its utilization in bones, inhibit bone loss, and manage osteoporotic or cardiovascular risks in menopausal women [53].

**Figure 2:**
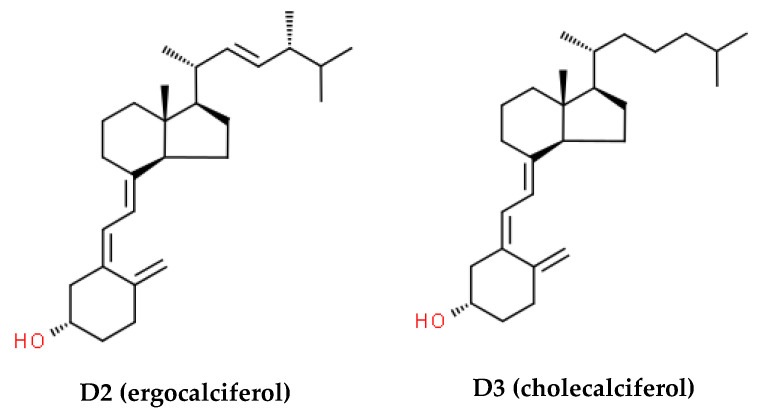
Chemical structures of vitamin D.

VitD is synthesized in the human skin through ultraviolet B light (UVB) exposure or obtained via dietary consumption as vitD2 (ergocalciferol) or vitD3 (cholecalciferol). These biologically inactive forms (Figure 2) are metabolized to 25-hydroxyvitD (25(OH)D) or calcidiol in the liver, then 25(OH)D is further hydroxylated by the kidneys to 1,25-dihydroxyvitD (1,25(OH)2D) or calcitriol, the bioactive vitD hormone.

The active form has multiple roles beyond intestinal calcium absorption and mineral homeostasis, as many cell types that are located in various tissues are known to express the vitD receptor (VDR) [54,55].

VitD deficiency is relatively common, as only a small percentage of the recommended daily vitD intake can be consumed even from the richest dietary sources (fatty fish, fish oil, egg yolks). Hence, vitD supplementation is essential to maintain the serum level of 30 ng/mL (75 nmoL/L) 25(OH)D needed for bone health. Higher serum levels of 40 to 60 ng/mL (100 to 150 nmol/L) are associated with the lowest risk of CVD and all-cause mortality [56]. Normal-weight adults can achieve the mentioned circulating levels via the daily ingestion of 2000 to 5000 IU, as individual responses to vitD supplementation vary considerably based on many factors, such as genetics, weight, health status, or medications [57].

**Figure 3:**
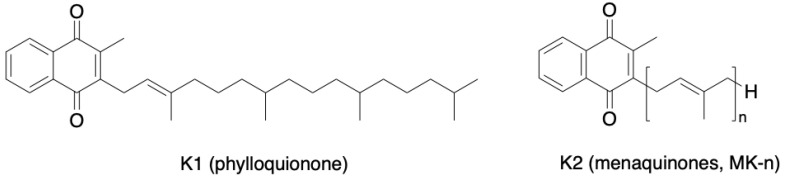
Chemical structures of naturally occurring vitamin K.

VitK is a fat-soluble vitamin, naturally occurring as vitK1 or phylloquinone and vitK2 or menaquinone (Figure 3).

Phylloquinone, found mostly in leafy dark-green vegetables, which are the major source of vitK intake, is absorbed in the jejunum and ileum and is stored mainly in the liver, while menaquinones (abbreviated as MK-n, where n stands for the number of isoprenyl units) are found in fermented foods and in animal products but can also be synthesized by the human intestinal microbiota and absorbed from the distal intestine [58]. Of the 12 different types of MKs (MK-4 to MK-15), the most common types in humans are short-chain MK-4 (which can be produced via the conversion of phylloquinone) and long-chain MKs, namely MK-7 to MK-10 [59]. VitK is an essential cofactor for the carboxylation of glutamic acid (Glu) in VKDPs, including osteocalcin (OC) and matrix glutamate (Gla) protein (MGP), that modulate bone metabolism and VC. VitK deficiency can reduce VKDPs’ activity and increase the risk of osteoporosis and fractures or VC and cardiovascular events [60]. Compared to phylloquinone, which is only 5–10% absorbed, vitK2 is almost completely absorbed and thus is the most used form for supplementation [61]. MK-4 is typically utilized in bone trials, whereas MK-7, with a higher bioavailability, is employed in trials with cardiovascular as well as bone health outcomes [62]. VitK, in particular vitK2, is known for its calcium homeostasis function and its deficiency seems to be responsible for the so-called “calcium paradox” phenomenon, defined by low calcium deposition in the bone and accumulation in the vessel wall [63].

### 3.2. Cardiovascular Health

#### 3.2.1. Vitamins D and K in Cardiovascular Health

CVD continues to be the leading cause of mortality worldwide and the leading cause of morbidity and mortality in postmenopausal women [64]. VC, a chronic inflammatory process mediated by proinflammatory cytokines including IL-1β, IL-6, TNF-α, and the nuclear factor kappa B (NF-κB) signaling pathway in both the intimal and medial layers of arterial walls, is a marker of CVD and an independent risk factor for stroke, myocardial infarction, and cardiovascular death [65]. Calcification was regarded for many years as a passive, degenerative, untreatable disease occurring in small and large arteries, but recent findings point to an active, complex condition that can be accelerated or inhibited by different molecules [66].

**Figure 4:**
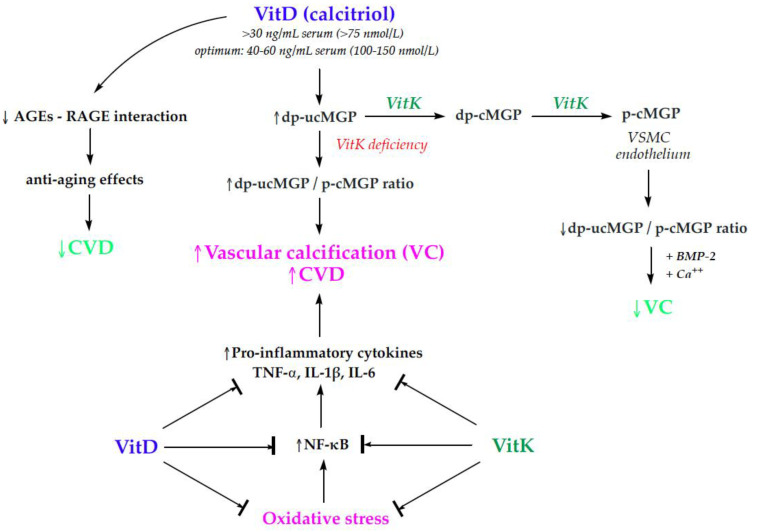
The impact of vitD and K on VC and CVD (AGEs—advanced glycation end products; BMP-2—bone morphogenetic protein 2; CVD—cardiovascular disease; dp-cMGP—dephosphorylated–carboxylated matrix Gla protein; dp-ucMGP—dephosphorylated–uncarboxylated matrix Gla protein; Gla—γ-carboxylated glutamic acid; IL—interleukin; NF-kB—nuclear factor kappa-light-chain enhancer of activated B cells; p-cMGP—phosphorylated-carboxylated matrix Gla protein; RAGE—receptor for advanced glycation end products; TNF-α—tumor necrosis factor-alpha; VC—vascular calcification; VitD—vitamin D; VitK—vitamin K; VSMC—vascular smooth muscular cells; ↑—increase; ↓—decrease).

VitK may alter the VC process through anti-inflammatory mechanisms and by enhancing the activity of MGP [67]. MGP, a small VKDP synthesized by the vascular smooth muscle cells and the endothelium, is the first protein recognized to inhibit VC and reverse the calcification process in the human body [68]. The bioactivation of MGP requires two vitK-dependent modifications, carboxylation and phosphorylation. Thus, reduced vitK levels are associated with dysfunctional species of MGP and VC in various populations, whereas high vitK levels can reverse this pathophysiological condition. The inactive forms of MGP, particularly dephosphorylated–uncarboxylated MGP (dp-ucMGP), a recognized marker for vascular vitK status, could be a potential biosignature for the assessment of calcification and cardiovascular risk, as well as microvascular health [69]. Interestingly, however, only vitD supplementation increases dp-ucMGP concentrations and causes a relative vitK deficiency, thus decreasing the MGP/dp-ucMGP ratio and MGP protection against VC [70] (Figure 4).

#### 3.2.2. Randomized Controlled Trials of Vitamins D and K in Cardiovascular Health

The characteristics and the main findings of the six selected studies [71,72,73,74,75,76] related to cardiovascular health are depicted in Table 2.

CAC and especially the progression of CAC is a strong predictor of acute myocardial infarction and cardiovascular mortality [77]. In addition, supplementation with vitK2 and D3 has been suggested to have a protective role in the progression of CAC [78]. In older healthy postmenopausal women participating in a three-year double-blind prospective RCT, the daily intake of phylloquinone plus a multivitamin formulation containing vitD3, calcium, and vitamins B, C, and E resulted in slower CAC progression compared to the control participants, who received the multivitamin formulation alone [72]. Consistent with these findings, in a randomized double-blind placebo-controlled trial, Braam et al. showed that a three-year supplementation with phylloquinone, vitD3, calcium, and magnesium had a beneficial effect on the elastic properties of the arterial vessel wall [71]. Both of these studies were of identical duration and had large sample sizes that fulfilled the power analyses without inherent biases. Given that the vitK dose in the Braam et al. study was twice as high (1 vs. 0.5 mg), with good compliance and an absence of adverse effects, there may be value in future studies examining the lowest possible dose for an effect. 

The intake of a fortified yogurt drink for 12 weeks increased circulating MK-7 and significantly decreased circulating ucOC and dp-ucMGP [73]. However, the markers of inflammation, endothelial dysfunction and lipid metabolism were not affected, which could be explained by the lower serum levels of these biomarkers in healthy menopausal women, minimizing the possibility of their reduction. The same team followed up with a second study [74], a single-blind randomized trial with a 6-week duration, where all tested products (fortified yogurts with VitD and/or MK-7 and capsules containing MK-7) significantly decreased serum undercarboxylated osteocalcin (ucOC) and plasma dp-ucMGP, reflecting bone and vascular vitK status improvement, respectively. The report concluded that yogurt fortified with vitamins K2 and D3 is a suitable matrix to improve the nutritional status of these fat-soluble vitamins [74]. Although the vitK dose was lower (28 vs. 58.3 μg MK-7) in their first study, the study duration was twice as long (12 vs. 6 weeks); therefore, the total amount of vitK consumed was roughly equal. These results were confirmed by a prospective cohort study with 644 community-dwelling adults assessed for over 13 years, where a low vitK status (high dp-ucMGP levels) was linked with lower handgrip strength, a smaller calf circumference, a lower functional performance score, as well as a higher frailty risk [79,80]. Moreover, data from two longitudinal analyses enrolling older participants from the Health, Aging and Body Composition Study showed that lower plasma dp-ucMGP levels were associated with better physical performance scores and a reduced risk of mobility disability [81,82]. Additionally, vitD and vitK co-supplementation for 12 months increased plasma adiponectin compared to the placebo, but did not change insulin sensitivity in osteopenic postmenopausal women [76]. 

Vignini et al. showed that taking extra virgin olive oil (VOO) enriched with vitK1, vitD3, and vitamin B6 for one year significantly decreased the levels of platelet aggregation, platelet NO, and high ROS in postmenopausal women compared to the placebo [75]. Moreover, the platelet membrane fluidity, which may be reduced in the estrogen-deficient state of menopause, increased after treatment. These results support the idea that a Mediterranean diet enhanced with vitamins K and D has antioxidant and anti-inflammatory properties and could prevent age-associated diseases, such as CVD and ischemic stroke, in menopausal women.

Oxidative stress and inflammation biomarkers were quantified in a RCT [83] among vitD-deficient women with polycystic ovary syndrome. After supplementation with vitD, vitK, and calcium (200 IU, 90 μg, and 500 mg, respectively, twice a day for 8 weeks), the values of plasma total antioxidant capacity (TAC) significantly increased and the malondialdehyde (MDA) levels, a key marker of lipid peroxidation, significantly decreased (*p* = 0.005 for all) compared to the placebo. The results demonstrate a positive balance between antioxidant mechanisms and prooxidant compounds [83].

Moreover, an observational prospective study with a 7-year follow-up in 601 persons (mean age: 70 ± 6 y, 50.4% women) showed that a combination of low vitD and vitK status was associated with adverse cardiovascular health and increased risk of all-cause mortality [84]. Similarly, a prospective study that followed 4742 participants for approximately 14 years, with a mean age of 52.6 ± 11.9 years and for a median of 14.2 years, concluded that a combined low vitD and vitK status was associated with an increased all-cause mortality risk [85].

### 3.3. Bone Health

#### 3.3.1. Osteoporosis and Vitamins D and K

As the world’s population ages, several conditions including sarcopenia, osteopenia and osteoporosis, frailty, and physical deterioration increase the risk of falls, which may lead to bone fractures. Recent findings in osteoporotic patients (95% women) have indicated that 98% of hip fractures and 100% of wrist fractures are caused by falls. Furthermore, hip fracture in the elderly is associated with a significant increase in mortality [86].

The results of a recent study suggest that vitD is associated with bone mass, a lower fracture risk, and can delay the aging process, as it regulates cell homeostasis and cellular senescence [87]. Moreover, vitD supplementation is a safe treatment that improves skeletal muscle and diabetes control, and lowers CVD risk and menopausal symptoms [88].

Rokidi et al. reported that postmenopausal women who sustained osteoporotic fractures had significant differences in their organic matrix quality compared to age- and BMD-matched controls without fracture incidents [89]. BMD is the strongest predictor of osteoporotic fracture risk; nevertheless, other parameters including markers of bone formation and resorption, the hydration of bone matrix, or changes in non-enzymatic crosslinks in bone collagen could also be used to assess bone health [90]. Several bone turnover markers (BTMs) including OC, alkaline phosphatase (ALP) or BAP, and the procollagen type 1 amino-terminal propeptide (P1NP), which could be used as bone formation markers, and the carbo-terminal telopeptide of type I collagen (CTX), urinary type-I collagen cross-linked-N-telopeptide (uNTX) and deoxypyridinoline (DPD), which could be used as bone resorption markers, are released during bone remodeling and reflect the bone metabolism [91].

#### 3.3.2. Randomized Controlled Trials of Vitamins D and K in Bone Health

The characteristics and the main findings of the studies related to bone health in apparently healthy, non-osteoporotic postmenopausal women who did not use any medications, vitamins and/or minerals known to influence bone metabolism [92,93,94,95,96,97,98,99,100,101,102,103,104,105] are depicted in Table 3.

After supplementation with VOO enhanced with vitK1, vitD3, and vitamin B6 for one year, postmenopausal healthy Italian women (*n* = 60) had lower ucOC and OC levels, reduced markers of oxidative stress, and an increased plasma total antioxidant capacity compared to baseline [103]. Moreover, a 12-week nutrition intervention with tailor-made Atlantic salmon containing high levels of vitD3 and vitK1 had a positive influence on bone health, as measured by bone biomarkers in healthy postmenopausal Norwegian women [104]. Both of these studies included women who were relatively young (~50–60 years of age), and the effects may vary in women who are older. Further, the results from these studies in white women cannot be extrapolated to other ethnic or minority groups.

A modified Mediterranean-style diet with a low glycemic index, supplemented with vitK1, vitD3, and bioactive phytocompounds (rho iso-alpha acids and berberine) for 14 weeks, resulted in a favorable bone biomarker profile with reduced serum OC in postmenopausal women with and without metabolic syndrome [98]. In this single-blind placebo-controlled pilot study, all women were instructed to engage in 150 min of weekly aerobic exercise, which may or may not have confounded the results. This also may make it more difficult to compare it to studies that do not incorporate physical activity or measure physical activity levels. Further analysis indicated that this treatment reduced the rate of resorption without a reduction in the rate of bone formation, creating a healthier bone metabolism [100]. 

Combined nutrition and lifestyle counseling interventions for 12 months increased the total BMD in healthy postmenopausal women, with a significantly higher BMD observed for the lumbar spine compared to the control [101]. Furthermore, the physical activity levels increased by more than 2000 steps per day in all three intervention groups, indicating that exercise behavior likely did not influence the results in this randomized study of 115 healthy postmenopausal women aged 55–65 years. In the same cohort of women, both vitK1 and MK-7 groups had a significantly lower serum ucOC/OC ratio, increased insulin-like growth factor 1 (IGF-1) levels, and reduced levels of urinary DPD, a bone resorption marker [102]. Similarly, in a large sample (*n* = 244) of healthy, non-osteoporotic women, with equal numbers of women recruited in four age groups (60–64, 65–69, 70–74, ≥75 yrs), who received combined vitamin K1 and D3 plus calcium for two years, a modest increase was shown in both the BMD and bone mineral content (BMC) at the site of the ultradistal radius but not at other sites in the hip or radius [94]. Race and ethnicity were not specified in either of these trials. 

Two trials noted that in healthy postmenopausal women who received phylloquinone and vitD3, the concentration of serum ucOC significantly decreased [83,84]. However, this treatment had no effect on bone turnover after 6 weeks in a cross-over double-blind study with a three-week washout period [83]. Of the 48 women recruited, there were 17 dropouts, which may have introduced some selection bias. This combination of phylloquinone and vitD3 also did not change the femoral neck, spine, and total-body BMD measurements in a 3-year double-blind RCT large sample of mostly white adults in the US whose treatment groups were well matched at baseline, except for in body weight and lumbar spine BMD [96]. Likewise, the double-blind placebo-controlled study of Binkley et al. concluded that a one-year supplementation of phylloquinone or MK4 combined with vitD and calcium reduced ucOC but did not change the BMD or bone turnover in healthy North American postmenopausal women [97]. The authors suggested that their results may have been influenced by the inclusion of only healthy women and the exclusion of osteoporotic women. Each of these studies appropriately excluded women on vitK antagonist medications (e.g., warfarin). Further, it appears that the duration of the treatment, namely 6 weeks, one year, and three years, led to the same conclusion.

Interestingly, one trial showed that six months of combined vitK2 and vitD intake increased the BMD of the lumbar spine in Korean women [87]. However, there was a significant dropout rate (~42%), which could have introduced selection bias, and there was no placebo group. Another study reported no changes in the BMD measurements of the lumbar spine after 3 years of supplementation with vitK1 and vitD [93]. This placebo-controlled trial did, however, result in a decreased loss of femoral neck BMD. Further, after 12 months of a single-blind RCT conducted in China with an excellent adherence rate and an appropriately powered sample size, the lumbar spine BMD decreased in the placebo group but not in the intervention (MK-7, D3, calcium) group. The bone loss in the femoral neck was significantly lower in the treatment groups compared to the placebo [105]. Thus, it appears possible that depending on the BMD measurement site (spine vs. femur vs. total body), there may or may not be a change after combined supplementation, and this should be taken into account when comparing studies. Furthermore, distinguishing differences between studies is also compounded by the use of different vitK supplementation. Specifically, the effects could be due to the sidechain of vitK2 and its length, or the fact that vitK2 is accumulated and used mainly in extra-hepatic tissues, while the liver is the main target tissue for vitK1 [106,107]. Yet, in patients with diabetes and chronic kidney disease, the combined intake of vitD and vitK1 from the diet and supplementation was associated with higher whole-body BMD, whereas a higher vitK1 intake (≥200 μg/day) was associated with an increased femoral neck BMD [108]. It is worth mentioning that, in addition to experimenting with vitK1 and MK-4, some trials used MK-7 knowing that this menaquinone has an extended half-life time and seems to be effective in much lower daily amounts compared to MK-4 (approx. 3 days vs. 1 h and 100 μg vs. 45 mg, respectively) [109].

The main findings and characteristics of the studies related to bone health in osteopenic or osteoporotic postmenopausal women [106,110,111,112,113,114,115,116,117,118,119] are depicted in Table 4.

Lower levels of estrogen in postmenopausal women increase the rate of bone resorption to bone formation, resulting in bone loss. Osteopenia, the initial stage of bone loss, which can progress to osteoporosis without treatment, is a common condition in postmenopausal women and is associated with an increased risk of fractures, morbidity, and mortality [120].

Combined treatment with vitK2 and vitD3 for two years preserves and increases the BMD in postmenopausal women with osteopenia and osteoporosis [112,113]. In early postmenopausal women, vitK2 cannot control the bone metabolic turnover but vitK2 and D3 co-treatment may control the activated bone turnover via suppressing bone resorption and sustaining the BMD [113]. Similarly, compared with vitK2 alone, joint vitamin administration significantly increased BMD via the stimulation of both bone formation and resorption [112]. Moreover, the combined therapy of vitamin D3 and vitamin K2 in osteoporotic postmenopausal women enhanced the increase in the BMD of the lumbar spine compared to a separate intake of vitD3 or vitK2 [106]. These outcomes may be explained by the fact that vitD3 induces osteocalcin and vitK2 is needed for its carboxylation [121].

A recent analysis [122] showed that a low BMD could be linked to the decreased absorption of fats and fat-soluble vitamins, such as vitamins D and K, due to low levels of taurine and bile acids. Taurine, a sulphur-containing non-proteinogenic β-amino acid with antioxidant protective effects, may be a marker of postmenopausal status and osteoporosis [123]. The study concluded that a combination of vitD, vitK MK-7, taurine, and calcium could be a synergistic treatment for osteoporosis. Also, another combination of vitamins K and D3, calcium, magnesium, and *Lactobacillus plantarum* maintained the total BMD in osteopenic menopausal women, while bone mass was significantly increased in the non-treated group [117].

The study by Douglas et al. in postmenopausal osteoporotic women with previous Colles fractures revealed that the intake of vitamins K and D corrected the ucOC, and that the carboxylation level became similar to that in premenopausal women [110]. Further, significant positive changes were noticed for all bone measurements [110]. In the carboxylated form, OC displays strong calcium-binding properties and is required for the proper mineralization of new bone [124]. Both K and D vitamins are required for OC synthesis, whereas vitK is necessary for its carboxylation [125]. An observational study reported that low vitK serum concentrations and high levels of ucOC are associated with a decreased BMD and higher fracture risk (especially hip fracture) [126]. The findings are in agreement with other studies in which vitamin K and D co-treatment increased the cOC levels and decreased ucOC [111] in postmenopausal women. Besides lowering ucOC, co-treatment with vitD and vitK may protect against fractures and cancers in postmenopausal women with osteopenia [114].

These results are in agreement with an RCT including postmenopausal Danish women with osteopenia, in which MK-7 treatment reduced both the serum levels of ucOC and the ucOC/OC ratio and, despite no significant BMD effects, maintained the microarchitecture of trabecular bone in the tibia [115]. Further analysis revealed that this treatment did not affect the biochemical markers of bone turnover, BMD, or bone microarchitecture [118].

A trial conducted in menopausal women with osteoporosis confirmed that the addition of vitK1 or vitK2 (MK-4) to vitD, calcium, and oral bisphosphonate only modestly effected hip geometry parameters [119]. Additionally, a cohort study that investigated the associations between vitamin K1 and D serum levels and hip fracture in an older population noticed a 50% higher hip fracture risk in participants with low levels of vitK1 and vitD compared with those with higher vitamin levels [127].

Although the findings indicate that supplementation with vitD, vitK, and calcium did not alter BMD after one year, Maria et al. analyzed whether the addition of melatonin and strontium to vitD3 and vitK2 could increase the lumbar spine BMD in postmenopausal osteopenic US women [116]. Melatonin is a nutritional supplement and chronotherapeutic hormone that may increase the bone density in menopausal women with osteopenia [128]. Strontium could increase the vertebral and femoral BMD and reduce fractures in postmenopausal osteopenic or osteoporotic women [129]. After one year, the vitamin K and D co-treatment with the addition of melatonin and strontium significantly increased the BMD of the lumbar spine and left femoral neck by 4.3% and 2.2%, respectively, with an upward trend for total left hip from baseline compared to the placebo. Moreover, the treatment decreased the 10-year probability of major osteoporotic fracture by 6.48% compared to a 10.8% increase in the placebo. Possible explanations may be the increased osteoblast differentiation and bone formation generated by increasing P1NP levels and decreasing the ratio of bone resorption to bone formation (↓CTX:P1NP), as well as the maintenance of healthy bone turnover by normalizing bone marker turnover towards equilibrium [116].

### 3.4. Findings and Recommendations

Our review presents evidence for the synergistic relationship between vitD and K co-administration and bone and cardiovascular health. The RCTs included in our comprehensive review varied greatly in their number of participants, from 20 to 440 postmenopausal women, in the duration of the study, from four weeks to three years, and in the geographical setting. Out of the 31 included studies, six of them were conducted in the United States, five in the Netherlands, four in Denmark and Japan, three in the United Kingdom, two in Greece and Italy, and one each in Canada, China, Korea, Norway, and Spain. Furthermore, the trials assessed various cardiovascular and bone health parameters including the elastic properties of the common carotid artery and the levels of ucOC, dp-ucMGP, NO, BMD, and BTMs (Table 5).

The RCTs also differed substantially in the vitD and K co-supplementation dosages given to the participants. The doses ranged from 50 IU to 4500 IU per day vitD3, while one trial used vitD2 supplement at a dose of 400 IU/day. Regarding vitK, both isoforms (vitK1 and vitK2) have been used as supplements. VitK1 was used in 15 studies in doses from 80 μg to 1 mg per day, and only in one study the dosage was 5 mg per day. Six studies used MK-4, 45 mg per day, and the remaining RCTs used MK-7 in doses ranging between 61 μg and 375 μg per day.

Considering vitD, both the International Osteoporosis Foundation and the Endocrine Society suggest targeting a circulating 25(OH)D level of 30 ng/mL in older adults, achievable with doses of 15 μg (600 IU) to 20 μg (800 IU) of vitD per day [130]. Nevertheless, new findings show that vitD deficiency frequently occurs in older individuals and is related to an increased risk of negative outcomes including falls, bone fractures, CVDs, and a lower quality of life [131]. New guidelines advocate for the use of higher doses of vitD supplements for the elderly, 4000 IU/day for those with a normal body weight and up to 10,000 IU/day for obese individuals, to reach and maintain a 25(OH)D concentration in the range of 30–50 ng/mL [132]. Previous studies have shown that higher doses of vitD supplementation (50,000 IU/week of ergocalciferol, 50,000 IU/week or 100,000 IU/month of cholecalciferol) can maintain 25(OH)D concentrations of 40–60 ng/mL with no signs of vitD toxicity [133]. Meanwhile, many studies have provided evidence showing that vitD is probably one of the least toxic fat-soluble vitamins, and the Endocrine Society and the Institute of Medicine have both agreed that acute vitD toxicity is extremely rare [132].

**Table 5:** Parameters assessed by the reviewed studies.

Parameter	Number of Studies	References
OC; cOC; ucOC; cOC/ucOC ratio	20	[73,76,92,94,95,96,97,98,99,100,102,103,104,105,110,111,113,114,115,118]
BMD	15	[93,94,96,99,101,102,103,105,106,112,113,114,116,117,118]
PINP, CTX; CTX/P1NP	4	[76,116,117,119]
IGF-1	3	[98,100,102]
MGP; dp-ucMGP	3	[72,74,134]
BAP	2	[113,118]
Adiponectin	1	[76]
CC; DC; IMT	1	[71]
DPD	1	[102]
NO; hROS	1	[75]
NTX	1	[97]

BAP—bone alkaline phosphatase; BMD—bone mineral density; CC—compliance coefficient; cOC—carboxylated osteocalcin; CTX—carbo-terminal telopeptide of type I collagen; DPD—deoxypyridinoline; dp-ucMGP—dephosphorylated–uncarboxylated MGP; hROS—highly reactive oxygen species; IGF-1—Insulin-like growth factor 1; IMT—intima–media thickness; MGP—matrix glutamate (Gla) protein; NO—nitric oxide; NTX—collagen cross-linked-N-telopeptide; OC—osteocalcin; P1NP—procollagen type 1 amino-terminal propeptide.

Despite the importance of vitK in human health, the optimal amount has still not been determined. Recent evidence has shown that women with vitK intakes of more than 100 μg/day, in line with the current vitK intake guidelines of approximately 90 and 120 μg/day for women and men, have a lower risk of falls and hip fractures compared to those with lower intakes (60 μg/day), independent of their vitD status and lifestyle factors (diet quality, smoking, physical activity) [135]. The administration of 45 mg of MK-4 daily was shown to be safe in postmenopausal women and did not create any thrombotic tendency. However, the administration of vitK could present some obstacles, as its absorption can be hindered by some factors including a low-fat diet and fat-blocking supplements, antibiotic use, bile acid sequestrants, gastrointestinal tract diseases, liver diseases, and estrogen drugs [136]. It is important to consider that women on long-term vitK antagonist (e.g., warfarin) therapy have an increased risk of systemic VC through the inhibition of MGP [69].

The randomized controlled studies performed on menopausal women of various ethnicities and health statuses suggest that combined vitD and vitK supplementation may be more beneficial for the prevention and potential treatment of age-associated diseases including CVD and osteoporosis than either supplementation alone. This approach may be part of a multifaceted strategy, which could include nutrition counseling and diet changing [137], addressing sedentary behavior and physical activity [138], avoiding tobacco exposure [139], and preventing or minimizing long-term glucocorticoid therapy [140], all holistic concepts needed to support the health of postmenopausal women.

### 3.5. Mechanisms of Action

Various preclinical and clinical studies [141] have previously identified possible mechanisms of action for cardiovascular and bone health management after vitamin D and K co-treatment that underscore the beneficial outcomes of the trials assessed in this review.

The synergistic relationship of vitD and K co-treatment is emphasized by the fact that the gene that encodes MGP, a calcification inhibitor VKDP, incorporates the binding sites of vitD in the MGP promoter regions [142]. The active MGP compared to inactive MGP or total MGP concentrations has been demonstrated to inhibit osteogenic differentiation, calcification, and the progression of CAC by binding to bone morphogenetic protein-2 (BMP-2) and reducing the mineralization of vascular cells [143]. Hence, through the downregulation of the BMP-2 pathway, carboxylated MGP could inhibit VC and osteoporosis [144]. Mechanistically, MGP can suppress and reverse the process of calcification by binding directly to the free calcium found in the blood vessels and hydroxyapatite crystals accumulated in the vessel walls [145]. It was previously shown that co-supplementation with vitD and vitK increased the production and the activation of MGP, significantly lowering circulating ucOC and dp-ucMGP and improving arterial stiffness and cardiovascular health in postmenopausal women [74,134]. Several studies have corroborated these outcomes. A longitudinal study enrolling 577 older adults showed that high plasma dp-ucMGP concentrations are linked with an increased CVD risk independent of other risk factors and vitD status [146]. Correspondingly, a recent meta-analysis showed that circulating dp-ucMGP is associated with an increased risk of cardiovascular and all-cause mortality [147]. Furthermore, several cross-sectional studies noted that increased dp-ucMGP levels are positively associated with arterial stiffness, a risk factor for CVD [148,149,150].

In menopause-related bone loss, an increased rate of bone turnover due to estrogen deficiency results in a remodeling imbalance, where the amount of bone formed by osteoblasts no longer matches the amount of bone removed by osteoclasts [151]. However, the combined administration of vitamins rebalances this process and improves the BMD [90]. It is well recognized that VitD intake enhances the production of OC, while vitK consumption increases the carboxylation rate of VKDPs, which is necessary for the proper formation, mineralization, and protection of bone tissue [101,106,112]. These processes have been confirmed by various bodies of evidence [152]. The increased affinity for calcium in cOC could lead to a higher proportion of OC being bound within the bone matrix, resulting in a decline in circulating total OC levels [118]. Notably, a meta-analysis including a total of 6759 participants concluded that vitK2 treatment improved the vertebral BMD and reduced the risk of fractures in osteoporotic postmenopausal women [153].

Several studies have revealed that vitD and K intake increase the osteoblastic activity and lower the ucOC/OC ratio [110,111]. In addition, in menopausal women with a previously low cOC and a low lumbar spine BMD, vitD and vitK supplementation increased the levels of cOC [92]. Also, the cOC/ucOC ratio was significantly enhanced in the supplementation groups [105]. Furthermore, the co-treatment reduced femoral neck bone loss [93] and significantly increased the BMD of the lumbar spine and left femoral neck, with a trend towards improvement in the total left hip [116].

Osteoblast mineralization was noticed after supplementation with vitK1, MK-4, and MK-7, which significantly improved the activity of ALP [154]. Correspondingly, MK-7 treatment increased the ALP activity, protein content, and OC production in osteoblastic cells, indicating that it can stimulate osteoblastic bone formation [155]. It has also been shown that MK-7 stimulates osteoblastogenesis and suppresses osteoclastogenesis by suppressing NF-κB activation through an increase in the inhibitor of nuclear factor kappa B (IκB) mRNA expression [156]. It is possible that vitK2 regulates osteogenic activity through the IL-6-mediated JAK/STAT signaling pathway by inhibiting STAT1 via Bcl-6 regulation [157]. Yet, vitK2 treatment increases the levels of ALP, OC, and runt-related transcription factor 2 (RUNX2), a master transcription factor for early osteogenic differentiation [124]. In addition, vitK2 stimulates autophagy in treated cells, increases the mRNA expression levels of ALP, OC, and RUNX2, and promotes osteogenic effects [158].

One of the main biological features of aging is increased inflammation, associated with the hyperactivation of osteoclastic bone resorption in postmenopausal osteoporosis [159]. The accumulation of senescent cells and the release of inflammatory markers such as TNF-α, IL-6, and IL-1 as a part of the senescence-associated secretory phenotype (SASP) cause a state of chronic low-grade systemic inflammation named inflammaging [160]. These pro-inflammatory cytokines, which can be produced by senescent osteoblasts and osteocytes, are partly regulated by aberrant NF-κB signaling; thus, inhibiting NF-κB could be a possible mechanism to address the SASP as an anti-inflammation therapy [161]. VitD has been shown to control inflammation, inhibit the translocation of NF-kB, and decrease endothelial dysfunction [162], while MK-7 may downregulate the expression of TNF-α, IL-1α, and IL-1β in a dose-dependent manner [163].

Furthermore, daily supplementation with VOO enriched with vitamins D3, K1, and B6 had an antioxidant effect by scavenging reactive oxygen and nitrogen species and prevented oxidative stress in postmenopausal women [75]. Dietary supplementation with vitaminized olive oil reduced oxidative stress markers including thiobarbituric acid reactive substances (TBARSs) and lipid hydroperoxides [103]. Countering oxidative stress and chronic inflammation using various antioxidants appears to be a major contributor to healthy aging [164]. Indeed, treatment with MK-7 and vitD could have an anti-inflammatory effect and reduce the risk of atherosclerosis by increasing plasma adiponectin levels [76]. In agreement with these findings, VK treatment increased OC gene expression, as well as adiponectin production [165]. Adiponectin, an insulin-sensitizing and anti-inflammatory adipokine, exerts anti-oxidative and vasoprotective properties [166]. Adiponectin can also activate AMP-activated protein kinase (AMPK) and peroxisome proliferator-activated receptor-alpha (PPAR-α), which are implicated in cellular energy homeostasis and have anti-inflammatory and anti-thrombotic actions [167]. An adequate level of VitD also diminishes oxidative stress and cardiovascular risk and prevents osteogenic trans-differentiation caused by the interaction between advanced glycation end-products (AGEs) and their receptor (RAGE) [168,169].

Other studies have examined OPG, the receptor activator of NF-κB ligand (RANKL), and RANK as another pathway. Sustained OPG release from vascular endothelial cells in response to inflammatory cytokines suggests that the OPG/RANKL/RANK axis could play a modulatory role in VC and inflammation [170]. OPG, a decoy receptor for RANKL, inhibits RANK–RANKL interactions, hence suppressing osteoclastogenesis and bone resorption [171]. Data from two clinical trials suggest that a modified Mediterranean-style diet supplemented with vitD3, vitK1, and berberine could regulate the OPG/RANKL/RANK axis [98,100]. The study of Kanellakis et al. also targeted this pathway as a therapeutic approach in osteoporosis management [102]. VitK2 consistently downregulated osteoclast differentiation and bone resorption, exerting anti-osteoclastogenic effects via increases in the OPG/RANKL ratio [172]. In preclinical studies, both vitK1 and vitK2 supplementation have supported osteogenesis by increasing the OC and OPG levels in diet-induced obese mice [173]. Also, in ovariectomized mice, a murine model that mimics menopausal osteoporosis, MK-4 inhibited RANKL signaling and decreased nuclear factor of activated T-cells 1 (NFATc1), osteoclast-associated receptor, and cathepsin K mRNA expressions [174]. Similarly, in ovariectomized female Sprague Dawley rats, bone loss was significantly reduced in the combined vitK and vitD supplementation group compared to the control [175]. In addition, MK-7 intake improved mechanical recovery and the bone microstructure, characterized by a greater bone volume in ovariectomized rats [176], while vitK1 and MK-4 supplementation reduced bone loss by modulating the osteoblast and osteoclast activities in high-fat-diet-induced obese mice [173]. VitK1 and MK-4 treatment was also shown to stimulate osteoblastogenesis in bone marrow cells and inhibit osteoclast formation by decreasing the expression of RANKL/osteoclast differentiation factor (ODF) and enhancing the expression of OPG/osteoclast inhibitory factor (OCIF) [177]. Interestingly, both MK-4 and MK-7 inhibited osteoclastogenesis by downregulating RANKL-induced NF-κB activation and stimulated osteoblastogenesis by ameliorating the suppression of TNF-α of Smad signaling; however, compared to MK-4, the effect of MK-7 was much stronger, thus requiring lower doses [156]. Additionally, vitD3 [178] and vitK2 treatment promoted osteogenesis effects through the activation of the Wnt/β-catenin signaling pathway [179,180].

Furthermore, the 25(OH)D concentration is considered to be essential for bone metabolism homeostasis. Some studies [92,102] reported significantly higher levels of 25(OH)D after co-supplementation with vitamins D and K, potentially leading to a lower fracture risk. Also, 25(OH)D may have anti-apoptotic effects in osteoblasts through the nongenomic activation of a VDR/phosphatidylinositol 3 kinase (PI3K)/Akt survival pathway that includes the phosphorylation of multiple p-Akt substrates and a reduction in caspase activities [181]. These results agree with previous reports showing that vitD3 could protect against apoptosis through the activation of the MEK/extracellular signal-regulated kinase (ERK) and PI3K/Akt survival pathways [182].

**Figure 5:**
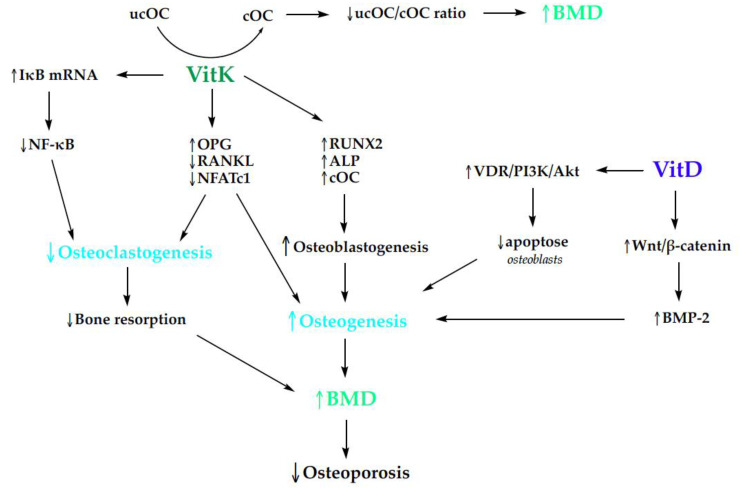
The impact of vitamins D and K on bone mineralization (Akt—protein kinase B; ALP—alkaline phosphatase; BMD—bone mineral density; BMP-2—bone morphogenetic protein 2; cOC—carboxylated osteocalcin; IκB—inhibitor of nuclear factor kappa B; NFATc1—nuclear factor of activated T-cells 1; NF-kB—Nuclear factor kappa-light-chain enhancer of activated B cells; OPG—osteoprotegerin; PI3K—Phosphatidylinositide-3-kinase; RANKL—the receptor activator of NF-κB ligand; RUNX2—runt-related transcription factor 2; ucOC—undercarboxylated osteocalcin; VDR—vitamin D receptor; VitD—vitamin D; VitK—vitamin K; ↑—increase; ↓—decrease).

Other mechanisms underlying the enhanced osteogenesis induced by vitD3 and vitK2 combined treatments are through the activation of the SIRT1/FOXO1 and AMPK/SIRT1 signaling pathways [183,184]. Co-treatment modulates the expression of genes involved in bone formation and mineralization including the induction of OC, as well as vascular endothelial growth factors (VEGFA) along with its receptor fms-related tyrosine kinase 1 (FLT1) [185]. Several potential molecular action mechanisms of vitD and vitK that affect bone metabolism are described in Figure 5.

Emerging evidence suggests that alterations in the gut microbiota (GM) through the regulation of nutrition and the use of vitamins including vitD and vitK could be novel mechanisms in cardiometabolic and bone health [45,186]. Recent studies have revealed that vitD3 supplementation ameliorates the gut’s microbial population [187], while vitK2 increases the number of *Bacteroides* species, part of the healthy human gut microbiome [188]. Similarly, vitD intake modulates GM via increasing *Bacteroidetes* and decreasing *Firmicutes* species [189]. For example, small intestinal bacterial overgrowth (SIBO), and intestinal microbial dysbiosis, is associated with altered vitK2 production by GM. Patients with SIBO presented higher dp-ucMGP serum levels, increased arterial stiffness and a higher risk of atherosclerosis and CVD [190].

### 3.6. Strength and Limitations

Our study adds important information in the field of nutrition and aging and has specific strengths. This is the first comprehensive review of RCTs to evaluate the combined effects of vitD and vitK in menopausal women. RCTs can attain adequate control over subjective biases and render a compelling indication that the treatments and actions cause an effect on human health. Another significant strength was the use of information from heterogeneous samples of populations from around the world, hence providing some degree of applicability to various ethnic groups. The majority of the included trials had appropriate sample sizes and control groups, and were in general rigorously controlled. Although the methods varied in terms of the delivery of the vitamins (fortified yogurt, extra virgin olive oil, oral supplementation), the studies were carefully designed and conducted and do not appear to have selection bias, but some attrition biases were noted.

Although the studies included in our comprehensive review were RCTs, some limitations could be considered in future trials, such as the following: the quality of the included trials was uneven and some biases were still present; the small sample sizes of several trials could have precluded definitive conclusions; the follow-up time was different; the intervention period was relatively short in a few cases; and physical activity, which can benefit bone health, was not always monitored.

## 4. Conclusions

Our narrative review synthesizes scientific findings, emphasizing the synergistic effects of two essential nutrients, vitD and vitK, with a primary focus on menopausal women. It encompasses 31 RCTs investigating both the vitD and vitK status and the supplementation of both vitamins. The review targets two significant age-related health concerns in menopausal women: CVD and osteoporosis.

Assessing the findings of these clinical studies, we demonstrate that vitD and vitK co-treatment offers antioxidant and anti-inflammatory benefits, modulates the bone metabolism, maintains bone health, and reduces CVD risks in postmenopausal women. The vitamins work synergistically to neutralize free radicals, lower inflammation, enhance calcium absorption, and activate the proteins essential for bone strength. Additionally, these vitamins support heart health by preventing blood vessel calcification and maintaining healthy blood pressure levels. This co-treatment provides a comprehensive approach to improving overall health in postmenopausal women.

As our understanding grows regarding the potent synergy of vitamins D and K, it reinforces the importance of maintaining a healthy diet that includes a variety of foods, such as fatty fish, vegetables and fermented dairy, to support both bone and cardiometabolic health. However, as it may be difficult for older people to acquire all their nutrients from dietary sources, there could be essential roles for vitamin supplements together with healthy eating.

Our findings improve the research base of these promising components for healthy aging. The dissemination of the data should promote the further exploration of menopausal symptoms and help women and health professionals make timely prevention and management decisions.

Future research could aim to answer some of the following questions: Could vitD and calcium supplementation along with lower doses of vitK induce tissue calcification? What are the possible long-term outcomes of combined high-dose vitD and vitK supplementation? Could vitD and vitK co-treatment suppress the impairment of the lungs caused by viral infections? What are the synergistic effects of combined vitD, vitK, and zinc supplementation?

## Figures and Tables

**Table 1:** Inclusion/exclusion criteria for selecting potential articles for the final review.

Inclusion Criteria	Exclusion Criteria
The study was published in a peer-reviewed scientific journal	The study was published in commentaries, case reports, books, dissertations, editorials, and conference proceedings
	The study was a review
The study utilized clinical trial study design	Not randomized clinical trial
The study aimed to examine the effects of vitamin D and vitamin K co-treatment	Not examining the co-treatment of vitamin D and vitamin K supplements
English (abstract)	Participants not experiencing menopause
	Not Human
	Age group under 50 years

**Table 2:** Characteristics of included randomized controlled trials related to cardiovascular health.

No.	Study, Year, Ref.	Country	Population Age	Duration Type of Study	Intervention	Comparison/Diets	Outcomes
1	Braam et al., 2004 [71]	The Netherlands	181 PM women of which 150 completed the study; analysis performed on 108 participants	3 years, double-blind, parallel, placebo-controlled intervention study	The participants were divided into 3 groups that received a daily supplement: (1) placebo (control group) (*n* = 40, 54.1 ± 3.0 y) (2) MD group: 500 mg calcium, 150 mg magnesium, 10 mg zinc, and 8 μg vitD/day (*n* = 30, 55.9 ± 2.8 y) (3) MDK group: the same formulation + 1 mg K1/d (*n* = 38, 55.4 ± 2.8 y)	The elastic properties of the common carotid artery were measured (including CC, DC, IMT, E)	After 3 years of supplementation and after adjustment for baseline heart rate, mean arterial pressure, age, weight and smoking: ↓ DC (8.8%, 95% CI: 1.9 to 21.4; *p* < 0.05) and CC (8.6%, 95% CI: 1.8 to 20.3; *p* < 0.05) in MDK group vs. placebo; ↑ Pulse pressure (6.3%, 95% CI: 0.7 to 17.1; *p* < 0.05) and E (13.2%, 95% CI: 5.3 to 35.8; *p* < 0.01) in MDK group vs. placebo; - no significant differences between the MD group and placebo for all parameters, as well as between the three groups for the change of IMT
2	Shea et al., 2009 [72]	USA	388 healthy men and PM women (60–80 y)	3 years, double-blind study	(1) Multivitamin with 500 μg phylloquinone/d (treatment) (*n* = 200, 68 ± 6 y) (2) multivitamin alone (control) (*n* = 188, 68 ± 5 y)	CAC progression	In a subgroup analysis of the participants who were adherent to supplementation (≥85%, *n* = 367): ↓ CAC progression in the phylloquinone group vs. control group (*p* = 0.03); In a subgroup analysis of the participants with preexisting CAC (Agatston score > 10 at baseline): ↓ CAC progression in the phylloquinone group at 6% vs. control group (*p* = 0.04); ↑ serum MGP in the phylloquinone group and ↓ in the control group (treatment effect: *p* ≤ 0.03 in all analysis)
3	Knapen et al., 2015 [73]	The Netherlands	39 healthy men and 26 PM women, mean age 56 ± 5 y (45–65 y)	12 weeks	(1) Standard yogurt (250 mL/container) (*n* = 29), 2x/day (2) Fortified yogurt (*n* = 27) with K2 (28 μg MK-7), n-3 PUFA, vitD, vitamin C, Ca, and Mg, 2x/day	Comparison between the groups at baseline and after 12 weeks	↑ circulating MK-7 levels (from 0.28 to 1.94 ng/mL) (*p* = 0.004 vs. standard yogurt) ↓ serum ucOC levels (*p* = 0.001) and plasma dp-ucMGP levels (*p* < 0.0001) vs. standard yogurt
4	Knapen et al., 2016 [74]	The Netherlands	43 healthy men and 64 PM women, 45–65 y	Intervention period of 42 days followed by a washout period of 2 weeks	(A) Yogurt Kplus: yogurt fortified with (per 100 mL): MK-7, vitD3 and C (12 mg), Mg (56 mg), Ca (108 mg), n-3 PUFA (40 mg), and fish oil (*n* = 36) (B) Yogurt K: yogurt fortified with only MK-7 (*n* = 34) (C) Capsules: soft gel capsules containing only MK-7 (*n* = 37)	The plasma MK-7, dp-ucMGP and ucOC were quantified and compared between the groups at baseline and after 14, 28, and 42 days - plasma MK-7 also determined at days 45, 49, and 56 (during the washout period)	Plasma MK-7 levels after 42 days: (A) 2.29 ± 0.08 ng/mL; (B) 2.17 ± 0.09 ng/mL; (C) 2.00 ± 0.09 ng/mL (*p* = 0.047 between the three groups) (mean ± SE) ↑ plasma MK-7 in (A) vs. (C) (*p* = 0.042) ↓ plasma MK-7 levels to 0.79 ± 0.05 ng/mL after 14 days of the washout period, being still higher compared with that at the start (*p* < 0.0001) ↓ plasma dp-ucMGP levels after 42 days: overall 445 ± 18 pmol/L (*p* = 0.005); (A) 485 ± 30 pmol/L; (B) 417 ± 33 pmol/L; (C) 434 ± 31 pmol/L (*p* = 0.019 between-group comparisons) ↓ ucOC levels after consumption of the yogurt products and the MK-7 capsules (*p* = 0.012), but w/o significant differences between groups
5	Vignini et al., 2017 [75]	Italy	60 healthy white PM women, 50–61 y	1 year, placebo-controlled trial	Oral supplementation with: (1) VitVOO group: 20 mL/d VOO fortified with D3 (50 μg/100 mL), K1 (0.70 mg/100 mL), and B6 (6.0 mg/100 mL); (2) PlaVOO group: only 20 mL/day VOO as placebo	Comparison between the groups at baseline and after 1 year	After 1 year of supplementation: ↓ NO levels in Vit VOO group vs. PlaVOO (37.20 ± 3.2 vs. 42.59 ± 4.31 nmol/mg protein; *p* < 0.001) ↓ hROS levels in Vit VOO group vs. PlaVOO (159.24 ± 15.3 vs. 226.23 ± 21.57; *p* < 0.05) ↑ plasma Na+/K+-ATPase activity in Vit VOO group vs. PlaVOO (0.650 ± 0.073 vs. 0.411 ± 0.043 µmol Pi/mg protein; *p* < 0.001) ↓ anisotropy in Vit VOO group vs. PlaVOO (0.152 ± 0.015 vs. 0.208 ± 0.025 arbitrary fluorescence numbers, *p* < 0.001); (0.204 ± 0.011 vs. 0.240 ± 0.014 arbitrary absorbance numbers, *p* < 0.001)
6	Rønn et al., 2021 [76]	Denmark	142 PM women with osteopenia, 63–73 y	12 months, placebo-controlled trial	(1) MK-7 group (*n* = 71): 375 μg MK-7, 800 mg Ca, 38 μg vitD3 daily (2) placebo group (*n* = 71): 800 mg Ca, 38 μg vitD3 daily	Comparison between the groups at baseline and after 3, 6, and 12 months	↓ ucOC in the MK-7 group (−70.3 (−75.6; −63.8)%) vs. placebo (−7.2 (−15.9; 2.0)%) after 12 months (*p* < 0.01) ↑ P-adiponectin in the MK-7 group (6.1 ± 20.1%) vs. placebo group (−0.7 ± 15.5%) after 12 months (*p* = 0.03) HOMA-IR and p-leptin—no changes

CAC—coronary artery calcification; CC—compliance coefficient; DC—distensibility coefficient; E—the Young’s Modulus; IMT—intima-media thickness; HOMA—IR-Homeostatic Model Assessment for Insulin Resistance; hROS—highly reactive oxygen species; MGP—matrix Gla protein; MK-7—menaquinone-7; NO—nitric oxide; OC—osteocalcin; PM—postmenopausal; ucOC—uncarboxylated osteocalcin; VOO—extra virgin olive oil; ↑—increase; ↓—decrease.

**Table 3:** Characteristics of included randomized controlled trials related to bone health in non-osteoporotic postmenopausal women.

No.	Study, Year, Ref.	Country	Population Age	Duration Type of Study	Intervention	Comparison/Diets	Outcomes
1	Schaafsma et al., 2000 [92]	The Netherlands	Healthy PM Dutch women (>5 y PM) with documented normal (*n* = 96) and low BMD (*n* = 45), 50–77 y	1 year	(1) Women with normal BMD (a double-blind study): Group A: 400 IU D3 and 80 μg K1; Group B: 80 μg K1; Placebo: 2.5 g of skimmed milk powder (2) Women with low BMD (an open study): Group C: 350 IU D3 and 80 μg K1; Group D: 400 IU D3	- Comparison vs. baseline and between the groups, at 3, 6, and 12 months; - All women (except placebo group) received about 1000 mg of additional calcium (total daily calcium intake of 2200 ± 2300 mg)	↑ 25(OH)D serum level after 3 months (*p* ≤ 0.0001), 6 months (*p* ≤ 0.0001), and 12 months (*p* < 0.001) in group A vs. group B + placebo, as well as vs. baseline (*p* < 0.005) ↑ 25(OH)D serum level after 1 y of supplementation with D3: 33 ± 29% (95% CI, 24.8–41.8%) and 68 ± 58% (95% CI, 50.1–84.6%) in women with normal and low BMD, respectively ↓ maximum 25(OH)D level: 29% in women with low BMD during supplementation with D3 in winter ↑ % cOC after 6 months (*p* = 0.009) and 12 months (*p* = 0.001) vs. placebo, and after 3, 6, 12 months (*p* ≤ 0.0001) vs. baseline in women with normal BMD after K1 supplementation (group A + B) ↓ % cOC at baseline for women with low BMD (of the lumbar spine and femoral neck, *p* < 0.005) vs. women with normal BMD; the difference disappeared after 1 y of supplementation with vitK1: 68 + 11% (95% CI, 64.5 ± 71.2%) vs. 72 + 6% (95% CI, 70.1 ± 72.9%), respectively
2	Braam et al., 2003 [93]	The Netherlands	181 healthy PM women (50–60 y), 155 completed the study	3 years, double-blind, parallel, placebo-controlled intervention study	The participants were divided into 3 groups that received a daily supplement: (1) placebo (control group) (*n* = 60, 54.6 ± 2.8 y); (2) MD group: 500 mg calcium, 150 mg magnesium, 10 mg zinc, and 8 μg vitD/day (*n* = 46, 55.7 ± 2.9 y); (3) MDK group: the same formulation + 1 mg K1/day (*n* = 56, 55.3 ± 2.8 y)	- Usual diets for all participants with supplements containing calcium, vitD, or vitK avoided throughout the study; - Comparison between the treated groups after 1, 2, and 3 years of supplementation	↑ BMD in femoral neck in MDK group vs. placebo and MD group (*p* < 0.05) after 3 years of supplementation ↓ Bone loss of the femoral neck with 1.7% (95% Cl: 0.35–3.44) in the MDK group vs. placebo and with 1.3% (95% Cl: 0.10–3.41) vs. MD group, respectively, after 3 years of supplementation and adjustment for baseline BMD, age, BMI, and years since menopause - No significant differences in BMD at the site of the lumbar spine among the three groups after 3 years of supplementation
3	Bolton-Smith et al., 2007 [94]	UK	244 healthy PM Scottish women, 60 y	2 years, double-blind, placebo-controlled trial	(1) placebo (*n* = 56 (61 at recruitment), 67.8 ± 6.0); (2) 200 μg/day K1 (*n* = 54 (60 at recruitment), 67.7 ± 4.9); (3) 10 mg (400 IU) D3 + 1000 mg calcium/day (*n* = 50 (62 at recruitment), 69.4 ± 6.4); (4) combined K1 + D3 and calcium (*n* = 49 (61 at recruitment), 67.8 ± 5.4)	- Comparison between the treated groups at baseline and after successive 6-month intervals of supplementation	↑ BMD and BMC at the site of the ultradistal radius in combined group (4) vs. baseline (*p* < 0.01) after 2 years of supplementation ↑ serum vitK1 by 157% (95% CI, 101, 212) (*p* < 0.001) after 2 years of vitK supplementation ↓ ucOC by 51% (95% CI, −47.5, −54.0) (*p* < 0.001) after 2 years of vitK supplementation ↑ serum 25-hydroxyvitD [25(OH)D] by 17% (*p* < 0.001), and ↓ PTH by 11% (*p* = 0.049) after 2 years of vitD supplementation
4	Bügel et al., 2007 [95]	Denmark	48 healthy PM women, 31 (62.5 ± 4.0 y) completed all three intervention periods	3 × 6-week, cross-over study, with 3-week washout periods	Usual diet supplemented with 0 (placebo), 200, and 500 μg phylloquinone (vitK1)/day; - all volunteers received 10 μg D3/day throughout the study period	- Comparison between the treated groups after each 6-week period of K1 supplementation	↑ daily K1 intake = ↑ cOC serum level and ↓ ucOC serum level, respectively, in a dose-dependent manner (*p* < 0.001) ↑ K1 serum level (*p* < 0.001) for 500 μg phylloquinone/day vs. placebo or 200 μg phylloquinone/day supplementation periods (which did not differ) (*p* = 0.15) ↑ serum total OC level (*p* < 0.001) for 500 (but not 200) μg phylloquinone vs. placebo
5	Booth et al., 2008 [96]	USA	452 healthy, ambulatory men and PM women (60–80 y), which 401 participants completed the trial	3 years, randomized, double-blind, parallel controlled trial	Diet supplemented with calcium (600 mg elemental calcium/day) and vitD (400 IU/day) containing either: (1) 500 μg phylloquinone (K1)/day (men: *n* = 95, 69 ± 5 y; women: *n* = 134, 68 ± 6 y); (2) no phylloquinone (men: *n* = 90, 69 ± 6 y; women: *n* = 133, 68 ± 5 y)	- Comparison between treatment groups at baseline and after 3 years of supplemented diet	↑ plasma phylloquinone concentrations (*p* < 0.0001) and ↓ % ucOC (*p* < 0.0001) after 3 years of vitK supplementation vs. the group that did not receive phylloquinone - overall increase in 25-hydroxyvitD plasma levels (*p* < 0.001) and a decrease of 1,25- dihydroxyvitD levels (*p* < 0.001) after 3 years of a daily supplement of 400 IU vitD, with the exception of women in the non-vitamin-K-supplemented group - No differences in lumbar spine or whole-body BMD between the two groups (*p* = 0.98 and 0.81, respectively) after 3 years of supplemented diet
6	Binkley et al., 2009 [97]	USA	381 healthy PM women	1 year, double-blind, placebo-controlled study	Phylloquinone (1) mg daily) (2) MK4 (45 mg daily), or (3) placebo - all participants received daily 315 mg calcium and 200 IU D3 supplementation.	- comparison between the groups at baseline and 1, 3, 6, and 12 months for biochemical parameters, and at baseline, 6, and 12 months for BMD, respectively	↓ serum ucOC after both Phylloquinone (−61.1%, 95% CI: −65.5%, −56.1%; *p* < 0.0001) and MK4 (−60.7%, 95% CI: −65.1%, −55.8%; *p* < 0.0001) treatment after 1 year vs. placebo ↓ total OC after both Phylloquinone (−8.38%, 95% CI: −13.15%, −3.35%; *p* < 0.005) and MK4 (−5.65%, 95% CI: −10.5%, −0.54%; *p* < 0.005) treatment after 1 year vs. placebo - No effect of phylloquinone or MK4 on serum bone-specific alkaline phosphatase (BSALP) or n-telopeptide of type 1 collagen (NTX) as well as on lumbar spine or proximal femur BMD measurements
7	Holick et al., 2010 [98]	USA	32 healthy PM women (50–70 y)	14-week, single-blinded, 2-arm placebo-controlled pilot study	(1) Treatment group (200 mg hop rho iso-alpha acids, 100 mg berberine sulfate trihydrate, 500 IU D3, and 500 μg K1, twice daily) (*n* = 15, 57.7 ± 1.0 y) (2) placebo (*n* = 17, 58.4 ± 1.6 y)	- all women consumed a modified Mediterranean -style, low-glycemic-load diet and limited aerobic exercise; - comparison between the groups at baseline and at 10 and 14 months	↓ serum OC by 31% (*p* = 0.02) in the treatment group and ↑ by 19% (*p* = 0.03) in the placebo group vs. baseline at 14 weeks ↑ serum 25(OH)D by 13% (*p* = 0.24) in the treatment group and ↓ by 25% (*p* < 0.01) in the placebo group ↑ serum IGF-I in the treatment group at 14 weeks (*p* < 0.01)
8	Je et al., 2011 [99]	Korea	78 healthy PM women (> 60 y), 45 completed the study	6 months, randomized intervention study	(1) K group (*n* = 38, which 18 completed the intervention): 15 mg K2 (MK-4) (3x/day) + 400 IU vitD (1x/day) + 315 mg calcium (2x/day); (2) Control group (*n* = 40, which 27 completed the intervention): 400 IU vitD (1x/day) + 315 mg calcium (2x/day)	- comparison between the groups after 6 months of treatment	↑ L3 BMD in K group vs. contro group (0.01 ± 0.03 g/cm^2^ vs. −0.008 ± 0.04 g/cm^2^, *p* = 0.049) ↓ ucOC in K group vs. contro group (−1.6 ± 1.6 ng/dL vs. −0.4 ± 1.1 ng/dL, *p* = 0.008)
9	Lamb et al., 2011 [100]	USA	51 PM women with the metabolic syndrome (no osteopenia/osteoporosis), 35–70 y, 45 completed the study	14-week, single-blind, 2-arm placebo-controlled randomized trial	(1) Intervention arm (200 mg hop rho iso-alpha acids, 100 mg berberine sulfate trihydrate, 500 IU D3, and 500 μg K1, twice daily) (*n* = 23, 61.7 ± 1.4 y); (2) placebo arm (*n* = 22, 58.9 ± 1.3 y)	- all women consumed a modified Mediterranean -style, low-glycemic diet and limited aerobic exercise - comparison between the groups at baseline and at 10 and 14 months	↓ serum OC (25%) to 2.28 ± 0.19 nmol/L and to 2.48 ± 0.19 after 10 and 14 weeks, respectively, vs. 3.31 ± 0.23 nmol/L at baseline (both *p* < 0.001) in the intervention arm ↑ serum OC (21%) to 2.96 ± 0.27 nmol/L and to 3.43 ± 0.28 (*p* < 0.001) after 10 and 14 weeks, respectively, vs. 2.84 ± 0.23 nmol/L at baseline in the placebo arm - Statistical changes in serum OC between arms after both 10 and 14 weeks (*p* < 0.001) ↑ serum 25(OH)D by 23% (139.95 ± 8.26 nmol/L vs. 113.69 ± 7.26 nmol/L, *p* = 0.001) in the intervention arm and ↓ by 12% (79.97 ± 5.27 nmol/L vs. 90.75 ± 6.46 nmol/L, *p* = 0.03) in the placebo arm after 14 weeks - Statistical changes in serum 25(OH)D between arms after both 10 and 14 weeks (*p* < 0.01) ↑ serum IGF-I in the intervention arm after 14 weeks (*p* < 0.01)
10	Moschonis et al., 2011 [101]	Greece	115 healthy PM women	12 months, RCT, with nutrition and lifestyle counseling	(1) CaD group (*n* = 26): 800 mg calcium and 10 μg D3; (2) CaDK1 group (*n* = 26): 800 mg calcium, 10 μg D3, and 100 μg K1 (phylloquinone); (3) CaDK2 group (*n* = 24): 800 mg calcium, 10 μg D3, and 100 μg K2 (MK-7); (4) CO group (control) (*n* = 39): usual diet	Comparison between the groups at baseline and after 12 months	↑ BMD in all intervention groups (*p* < 0.001 vs. CO) after 12 months ↑ L2-L4 BMD in the CaDK1 and CaDK2 groups (*p* = 0.001 vs. CO) after 12 months
11	Kanellakis et al., 2012 [102]	Greece	115 healthy PM women (55–65 y, mean of 62.0 ± 5.8 y)	12 months, RCT, with nutrition and lifestyle counselling	(1) CaD group (*n* = 26): 800 mg calcium and 10 μg D3; (2) CaDK1 group (*n* = 26): 800 mg calcium, 10 μg D3, and 100 μg K1 (phylloquinone); (3) CaDK2 group (*n* = 24): 800 mg calcium, 10 μg D3, and 100 μg K2 (MK-7); (4) CG (control group) (*n* = 39): usual diet	All three intervention groups received calcium and vitamins in fortified dairy products; Comparison between the groups at baseline and after 12 months	↑ serum 25(OH)D levels in all intervention groups vs. CG (*p* = 0.01) ↑ serum IGF-I levels in the CaDK2 group vs. CG (*p* < 0.05) ↓ serum ucOC/OC ratio and ↓ urine deoxypyridinoline (DPD) levels for both CaDK1 and CaDK2 groups vs. CaD and CG groups (*p* = 0.001 and *p* < 0.05, respectively) ↑ total-body BMD for both CaDK1 and CaDK2 groups vs. CG (*p* < 0.05) ↑ L2-L4 BMD in the CaDK1 and CaDK2 groups vs. CG (*p* < 0.01)
12	Mazzanti et al., 2015 [103]	Italy	60 Caucasian healthy PM women, 50–61 y	1 year, randomized, placebo-controlled trial	Oral supplementation with: (1) VitVOO group: 20 mL/day VOO fortified with D3 (50 mg/100 mL), K1 (0.70 mg/100 mL), and B6 (6.0 mg/100 mL); (2) PlaVOO group: only 20 mL/day VOO as placebo	- Comparison between the groups at baseline and after 1 year	↓ ucOC levels in Vit VOO group vs. PlaVOO (2.60 ± 0.14 vs. 3.12 ± 0.19 ng/mL, *p* < 0.001) ↓ ucOC/carbOC ratio in Vit VOO group vs. PlaVOO (*p* < 0.05) ↑ BMD-T-score in Vit VOO group vs. PlaVOO after 1 year (−1.28 ± 0.18 vs. −2.43 ± 0.32, *p* < 0.05) ↓ oxidative stress biomarkers in VitVOO group vs. PlaVOO after 1 year: TBARS (12.10 ± 1.70 vs. 41.68 ± 3.68 nmol/mL, *p* < 0.001), lipid hydroperoxide (3.10 ± 0.30 vs. 8.8 ± 0.6 nmol/mg prot, *p* < 0.01) and conjugated diene (2.10 ± 0.30 vs. 4.5 ± 0.41, *p* < 0.01) plasma levels
13	Graff et al., 2016 [104]	Norway	122 healthy PM women, 55 ± 5 y	12 weeks, randomized intervention trial	Salmon groups (150 g/2x/week + 1000 mg Ca/day) fortified with: (1) HD/HK: 114 μg D3/162 μg K1 (*n* = 31); (2) LD/HK: 27 μg D3/162 μg K1 (*n* = 30); (3) HD/LK: 105 μg D3/9.6 μg K1/kg (*n* = 31); (4) Tablet group (800 IU D3 + 1000 mg Ca/day) (*n* = 30)	Comparison between the groups at baseline and after 12 weeks	↓ serum ucOC within the HD/HK (*p* < 0.001) and LD/HK (*p* = 0.026) groups vs. baseline, and in all treated groups vs. the tablet group (HD/HK group: *p* = 0.004; LD/HK group: *p* = 0.035; HD/LK group: *p* = 0.020) ↓ serum cOC within the tablet (*p* < 0.05) and HD/LK (*p* < 0.001) groups vs. baseline ↓ GLU/GLA ratio decreased within the HD/HK group (*p* < 0.001) vs. baseline and in all treated groups vs. the tablet group (HD/HK group: *p* = 0.001; LD/HK group: *p* = 0.025; HD/LK group: *p* = 0.003)
14	Zhang et al., 2020 [105]	China	311 healthy men and PM women, 50–75 y (59.78 ± 6.60 y)	12 month, single-blind RCT	(1) placebo group (*n* = 65, 61 at the end); (2) 50-K2 (50 µg MK-7/day) (*n* = 84, 79 at the end); (3) 90-K2 (90 µg MK-7/day) (*n* = 78, 74 at the end); (4) 90-K2-plus (90 µg MK-7/day), calcium (500 mg/day), and vitD3 (10 µg/day) (*n* = 84, 81 at the end)		After 12 months of intervention: ↓ BMD in the placebo group (−0.006 g/cm^2^; 95% CI − 0.017, 0.004), but not in the 90-K2-plus group at lumbar spine (0.002 g/cm^2^; 95% CI − 0.005, 0.009) ↓ the bone loss of femoral neck in PM women in the 90-K2 and 90-K2-plus groups (treatment × time, *p* = 0.006) vs. placebo, but no effects in men ↑ serum vitK2 increased in all the three treatment groups (50-K2 group: + 0.43 nmol/L; 90-K2 group: + 0.22 nmol/L; 90-K2-plus group: + 0.40 nmol/L) (treatment × time, *p* = 0.015) ↑ cOC/ucOC ratio in the intervention groups (treatment × time, *p* < 0.001) ↑ serum 25(OH)D of 90-K2-plus group after additional vitD3 supplementation (treatment × time, *p* = 0.013)

BAP—bone alkaline phosphatase; BMC—bone mineral content; BMD—bone mineral density; cOC—carboxylated osteocalcin; CTX—carbo-terminal telopeptide of type I collagen; dp—desphospho-; HX—hip fracture; IGF-I—Insulin-like growth factor I; MGP—matrix Gla protein; MK-7—menaquinone-7; OC—osteocalcin; P1NP—procollagen type 1 amino-terminal propeptide; PM—postmenopausal; PRE—pre-menopausal; RCT—randomized controlled trial; VOO—extra virgin olive oil; VX—vertebral fractures; ↑—increase; ↓—decrease.

**Table 4:** Characteristics of included randomized controlled trials related to bone health in osteopenic/osteoporotic postmenopausal women.

No.	Study, Year, Ref.	Country	Population Age	Duration Type of Study	Intervention	Comparison/Diets	Outcomes
1	Douglas et al., 1995 [110]	UK	20 osteoporotic PM women with previous Colles fractures, 52–73 y (mean of 61.7 y)	2 weeks followed by 4 weeks without vitamins; Crossover study controlled with placebo, with a washout period of 3 months	Group 1 (*n* = 10): K1 Group 2 (*n* = 10): K1 + D2 Doses: 1 mg K1/day (in the morning); 200 IU D2/day (in the afternoon or evening)	- Each group was its own control (vs. placebo); - Comparison of OC level between the study’s PM women and younger pre-menopausal (PRE) women on the staff (*n* = 10, 22–39 y, mean of 29,6 y); - Comparison of bone mass measurements with 2 community controls of same age (who have no specific role in the study)	↑ total serum OC after K1; higher level after K1 + D2 (*p* < 0.01) ↑ degree of carboxylation (*p* < 0.001 after K1; *p* < 0.01 after K1 + D2) at values comparable to those in PRE women (73% vs. 57%, the initial values) - The degree of carboxylation persists 4 weeks after K1 treatment (64%, *p* < 0.05), being completely lost at 14 weeks
2	Iwamoto et al., 2000 [106]	Japan	92 osteoporotic PM women (>5 y PM), 55–81 y	2 years, parallel study	D group (*n* = 29, mean of 63.4 y): D3 (1α hydroxyvitD3), 0.75 µg/day; K group (*n* = 22, mean of 65.8 y): K2 (MK-4), 45 mg/day; DK group (*n* = 21, mean of 63.6 y): D3 + K2; C group (*n* = 20, mean of 63.5 y): calcium (as calcium lactate), 2 g/day	- Comparison vs. baseline and between the groups, at 0, 1, and 2 years	↓ BMD in C group (*p* < 0.001) vs. baseline ↑ BMD in D group (*p* < 0.05) and K group (*p* < 0.001) vs. C group ↑ BMD in DK group vs. C, D, and K groups (*p* < 0.0001, *p* < 0.05, and *p* < 0.01, respectively)
3	Takahashi et al., 2001 [111]	Japan	43 PRE (22–52 y, 34.5 ± 10.3) and 48 PM (54–87 y, 74.4 ± 6.9) healthy females, 89 osteoporotic female patients (49–94 y, 73.5 ± 9.4) with vertebral fractures (VX), and 24 female patients (52–93 y, 79.9 ± 9.5) with hip fracture (HX)	4 weeks	56 of 89 VX patients were treated orally as follows: Group K2: 22 VX patients (56–81 y, 67.6 ± 6.8) received 45 mg K2(MK-4)/day; Group D3: 13 VX patients (61 ± 88 y, 72.1 ± 8.9) received 1 μg of 1α-hydroxyvitD3/day; Group K2 + D3: 21 VX patients (49 ± 88 y, 75 ± 9.2) received 45 mg K2 and 1 μg D3/day	- Comparison between the PRE, PM, VX, and HX groups; - comparison between the treated VX groups after 4 weeks of treatment and vs. baseline	↑ OC in PM (*p* < 0.001 vs. PRE) and in VX (*p* < 0.001 vs. PRE and *p* < 0.05 vs. PM) ↓ OC in HX than in PM (*p* < 0.05) and VX (*p* < 0.001) - UcOC was higher in PM, VX, and HX than in PRE, but not significantly - The ucOC/OC ratio was higher in HX (*p* < 0.05) than in PM and in VX ↓ ucOC in the groups K2 and K2 + D3 after 4 weeks of treatment with K2, D3, and K2 + D3 vitamins, respectively, in the 56 VX patients ↓ ucOC/OC ratio to approximately 80% after 4 weeks of treatment with K2 and K2 + D3 (only vitD3 did not decrease this ratio)
4	Ushiroyama et al. 2002 [112]	Japan	172 PM women with vertebral BMD of the lumbar spine < 0.98 g/cm^2^ (osteopenia and osteoporosis), of which 126 completed the study	2 years	All patients were divided into 4 groups (*n* = 43 for each group at the start of the study): K2 group (*n* = 30, 54.1 ± 7.4 y): MK-4, 45 mg/day; D3 group (*n* = 32, 52.8 ± 5.6 y): 1 μg/day vit D3 (1α- hydroxyvitD3) K2 + D3 group (*n* = 31, 53.3 ± 4.5 y): combined therapy; Control group (*n* = 33, 53.5 ± 6.0 y)	- Control group received dietary therapy alone; - BMD and the bone markers were measured after 0, 6, 12, 18, and 24 months of treatment; - Comparison between the treated groups after 4 weeks of treatment and vs. baseline	↑ BMD with 0.278 ± 6.55% and 0.135 ± 5.44% (both *p* < 0.05 vs. control) after 18 and 24 months of K2 treatment, respectively ↑ BMD with 4.10 ± 5.88%, 5.86 ± 6.85%, 5.01 ± 8.11%, and 4.92 ± 7.89% (*p* < 0.001 vs. control in all cases) after 6, 12, 18, and 24 months of combined therapy K2 + D3, respectively
5	Yasui et al. 2006 [113]	Japan	34 PM women (mean of 53 y) with BMD at the lumbar spine < 0.809 g/cm^2^ (osteopenia and osteoporosis), which 30 completed the study	2 years	Group K2: (*n* = 17, 52.9 ± 6.2 y) 45 mg K2/day, orally; Group K2 + D3: (*n* = 17, 54.9 ± 6.8 y) 45 mg K2 + vitD3 (1α-hydroxyvitD3) daily. One patient from group K2 and 3 patients from group K2 + D3 dropped out of the study	- Comparison between the treated groups before and at 1 and 2 years after the start of supplementation	↓ ucOC serum levels in group K2 at 1 year and in group K2 + D3 at 1 and 2 years after the start of supplementation (*p* < 0.05) ↓ intact OC and BAP serum levels only in group K2 + D3 at 1 and 2 years after the start of supplementation (*p* < 0.05) ↓ BMD in group K2 at 1 and 2 years (*p* < 0.05 and *p* < 0.01, respectively) BMD was sustained in group K2 + D3
6	Cheung et al., 2008 [114]	Canada	440 PM women with osteopenia, with a mean serum 25-hydroxyvitD level of 77 nmol/L at baseline	2 years, randomized, double-blind, placebo-controlled trial, extended to an additional 2 years for earlier participants	(1) 5 mg/day vitK1 (*n* = 217, 58,9 y (40.1–80.5), 198 completed the study at 2-y, 97 at 3-y, and 33 at 4-y) (2) placebo (*n* = 223, 59.2 y (46.1–82.3), 202 completed the study at 2-y, 107 at 3-y, and 40 at 4-y); - all participants received a daily diet supplemented with 1500 mg of calcium and 800 IU of vitD (diet plus supplements)	- Comparison between the two groups at baseline, 3, 12, 24, 36, and 48 months for serum parameters, and at baseline, 24, and 48 months, or at final visit, for BMD, respectively	↓ BMD by −1.28% and −1.22% (*p* = 0.84) (difference of −0.06%; 95% CI, −0.67% to 0.54%) at the lumbar spine and −0.69% and −0.88% (*p* = 0.51) (difference of 0.19%; 95% CI, −0.37% to 0.75%) at the total hip in the vitK and placebo groups, respectively, over 2 years - No significant differences in changes in BMD at any site between the two groups over the 2- to 4-y period ↑ K1 serum levels in the K1 group vs. Placebo (22.6 nmol/L vs. 2.0 nmol/L, *p* < 0.0001) at 2 years ↓ ucOC and ↓ % ucOC in the K1 group vs. placebo (−52.8% vs. −3.5%, *p* < 0.0001, and −21.4% vs. −2.0%, *p* < 0.0001, respectively) at 2 years - Fewer women in the vitK group had clinical fractures (9 vs. 20, *p* = 0.04) and fewer had cancers (3 vs. 12, *p* = 0.02)
7	Rønn et al., 2016 [115]	Denmark	142 osteopenic PM women, 60–80 y	12 months, double-blind, randomized, placebo-controlled trial	(1) MK-7 group (*n* = 71): 375 μg MK-7, 800 mg Ca, and 38 μg vitD/day; (2) placebo group (*n* = 71): only 800 mg Ca, and 38 μg vitD/day	- Comparison between the groups at baseline and after 3, 6, and 12 months	↓ ucOC in the MK-7 group −65.6% (59.1; 71.0) vs. placebo −6.4%(−13.5; 1.2) after 3 months (*p* < 0.01), remaining low throughout the study (*p* < 0.05) ↓ ucOC in the MK-7 group (−65.2 ± 23.5%) vs. placebo (−0.03 ± 38.5%) after 1 year (*p* < 0.01) - Trabecular number in tibia (−0.1 ± 1.9%), trabecular spacing (+1.2 ± 8.0%), and trabecular thickness (+0.2 ± 1.7%) were unchanged in the MK-7 group (vs. −3.5 ± 2.2%, +4.5 ± 9.7%, and +4.0 ± 2.2%, respectively, in placebo group) (*p* < 0.05 between-groups)
8	Maria et al., 2017 [116]	USA	23 osteopenic PM women, 49–75 y (mean of 58.6 ± 1.12 y), which 22 completed the study	1-year, double-blind, randomized, placebo-controlled trial	(1) MSDK group: 5 mg melatonin, 450 mg strontium (citrate), 2000 IU vitD3, and 60 μg vitK2, divided into two capsules/day; (2) placebo: identical capsules with plant fiber	- Comparison between the groups at baseline and after 12 weeks	↑ BMD in lumbar spine L1-L4 (4.3%) and left femoral neck (2.2%) in the MSDK group vs. placebo, with an upward trend for total left hip (*p* = 0.069) ↑ serum P1NP levels in the MSDK group vs. placebo (*p* ≤ 0.05 after 6 months and *p* ≤ 0.01 after 1 year) ↓ bone turnover (CTX/P1NP) in the MSDK group in a time-dependent mode ↑ mood and sleep quality in the MSDK group ↑ urinary melatonin-sulfate levels in the MSDK group vs. placebo (*p* = 0.0463) - Correlation between melatonin levels and lumbar spine BMD (*p* = 0.029, r = 0.487; 95% CI = 0.0566 to 0.7648)
9	Morato-Martínez et al., 2020 [117]	Spain	79 osteopenic PM women	6 months, randomized, parallel, double-blind clinical trial	(1) experimental group (EG) (*n* = 40, 33 at the end): an experimental enriched product (calcium, vitD, vitK (80 µg), vitamin C, zinc, magnesium, L-leucine and probiotic (*Lactobacillus plantarum* 3547)) (2) control group (CG) (*n* = 39, 32 at the end): the same product without enrichment	- Comparison between the groups at baseline and after 6 months	↑ bone mass in the EG group vs. the CG group (0.01 ± 0.03 vs. −0.01 ± 0.03 kg; *p* < 0.05) ↑ serum P1NP levels in the EG group vs. the CG group (13.19 ± 25.17 vs. −4.21 ± 15.62 ng/mL; *p* < 0.05) ↓ CTX in the EG group vs. the CG group (−0.05 ± 0.19 vs. 0.04 ± 0.14 ng/mL; *p* < 0.05) ↓ systolic and diastolic blood pressure in the EG group vs. baseline
10	Rønn et al., 2021 [118]	Denmark	119 PM women with osteopenia, 67.3 ± 4.4 y	3 years	(1) MK-7 group (*n* = 62): 375 μg MK-7, 800 mg Ca, and 38 μg vitD3/day; (2) placebo group (*n* = 57): only 800 mg Ca, and 38 μg vitD3/day	- Comparison between the groups at baseline and after 3, 6, and 12 months	↓ total OC by 9.1 ± 25.6% (*p* < 0.01) in the MK-7 group (remaining unchanged 2.1 ± 19.6% (*p* = 0.11) in the placebo group) - Significant interaction between treatment group and time for total OC after 3 years (*p* <0.01) ↑ P1NP by 15.2 ± 39.4% (*p* < 0.05) in the MK-7 group (remaining unchanged in the placebo group) ↑ CTX and vitD in both groups (*p* < 0.03 for all) ↓ BAP in both groups (*p* < 0.01 for both) ↓ BMD at total hip (by 1.5 ± 2.5% for MK-7 vs. 2.4 ± 2.7% for placebo) and lumbar spine (by 1.8 ± 3.9% for MK-7 vs. 1.1 ± 3.1% for placebo) in both groups (*p* < 0.02 for all), and at the femoral neck only in the MK-7 group (*p* < 001) (by 1.5 ± 3.5% for MK-7 vs. 1.0 ± 5.0% for placebo) - Changes in microstructure were similar between groups over 3 years
11	Moore et al., 2023 [119]	UK	105 PM women with osteoporosis and sub-optimum vitK status, 55–85 y (68.7 ± 12.3 y)	18 months	(1) vitK1 arm (1 mg/day) (*n* = 35, 32 at the end); (2) vitK2 arm (45 mg MK-4/day) (*n* = 35, 31 at the end); (3) placebo (*n* = 35, 30 at the end) - all three groups: standard treatment of oral bisphosphonate and calcium (1 g/day) and/or vitD (800 IU/day)	- Comparison between the groups at baseline and after 3, 6, 12, and 18 months	↑ trend in CTX and P1NP (*p* < 0.001) over time in all 3 arms Changes in HSA parameters at the intertrochanter (IT) and femoral shaft (FS): (a) significant interaction between baseline vitK1 and the endocortical diameter (*p* = 0.004) independently of the treatment arms (b) IT endocortical diameter (% change placebo: 1.5 [4.1], K1 arm: −1.02 [5.07], *p* = 0.04) (c) FS subperiosteal/outer diameter (placebo: 1.78 [5.3], K1 arm: 0.46 [2.23], *p* = 0.04) (d) FS cross sectional area (placebo: 1.47 [4.09], K1 arm: −1.02 [5.07], *p* = 0.03)

BAP—bone alkaline phosphatase; BMC—bone mineral content; BMD—bone mineral density; carbOC—carboxylated osteocalcin; CTX—carbo-terminal telopeptide of type I collagen; dp—desphospho-; DPH—1,6-phenyl-1,3,5-hexatriene; HOMA-IR—Homeostatic Model Assessment for Insulin Resistance; hROS—highly reactive oxygen species; HX—hip fracture; IGF-I—Insulin-like growth factor I; MK-7—menaquinone-7; OC—osteocalcin; P1NP—procollagen type 1 amino-terminal propeptide; PM—postmenopausal; PRE—pre-menopausal; RCT—randomized controlled trial; uc—undercarboxylated; VX—vertebral fractures; ↑—increase; ↓—decrease.

## Data Availability

Data are contained within the article.
